# Investigating the sex-dependent effects of prefrontal cortex stimulation on response execution and inhibition

**DOI:** 10.1186/s13293-021-00390-3

**Published:** 2021-08-17

**Authors:** Daniel J. Fehring, Ranshikha Samandra, Zakia Z. Haque, Shapour Jaberzadeh, Marcello Rosa, Farshad A. Mansouri

**Affiliations:** 1 Cognitive Neuroscience Laboratory, Monash Biomedicine Discovery Institute, Department of Physiology, Monash University, Melbourne, VIC 3800 Australia; 2 ARC Centre of Excellence in Integrative Brain Function, Monash University, Melbourne, VIC 3800 Australia; 3 Department of Physiology, Monash Biomedicine Discovery Institute, Monash University, Melbourne, VIC 3800 Australia; 4 Department of Physiotherapy, Non-Invasive Brain Stimulation & Neuroplasticity Laboratory, Monash University, Melbourne, VIC 3199 Australia

**Keywords:** Sex dependency of cognitive functions, Brain stimulation, Learning, Stop-Signal Task

## Abstract

Context-dependent execution or inhibition of a response is an important aspect of executive control, which is impaired in neuropsychological and addiction disorders. Transcranial direct current stimulation (tDCS) of the dorsolateral prefrontal cortex (DLPFC) has been considered a remedial approach to address deficits in response control; however, considerable variability has been observed in tDCS effects. These variabilities might be related to contextual differences such as background visual-auditory stimuli or subjects' sex. In this study, we examined the interaction of two contextual factors, participants' sex and background acoustic stimuli, in modulating the effects of tDCS on response inhibition and execution. In a sham-controlled and cross-over (repeated-measure) design, 73 participants (37 females) performed a Stop-Signal Task in different background acoustic conditions before and after tDCS (anodal or sham) was applied over the DLPFC. Participants had to execute a speeded response in Go trials but inhibit their response in Stop trials. Participants' sex was fully counterbalanced across all experimental conditions (acoustic and tDCS). We found significant practice-related learning that appeared as changes in indices of response inhibition (stop-signal reaction time and percentage of successful inhibition) and action execution (response time and percentage correct). The tDCS and acoustic stimuli interactively influenced practice-related changes in response inhibition and these effects were uniformly seen in both males and females. However, the effects of tDCS on response execution (percentage of correct responses) were sex-dependent in that practice-related changes diminished in females but heightened in males. Our findings indicate that participants' sex influenced the effects of tDCS on the execution, but not inhibition, of responses.

## Highlights

1. In a fully counterbalanced (for sex and all experimental conditions), sham-controlled cross-over study, we examined the effects of tDCS over the left DLPFC in the context of Stop-Signal task.
2. The effects of tDCS on response inhibition was uniform across both males and females.
3. The effects of tDCS on response execution differed in males and females.
4. The tDCS mainly modulated the practice-related (learning-related) changes in participants’ performance, but these effects of tDCS were different between males and females.
5. These findings highlight the need to adequately control for participants' sex and the need to develop sex-specific tDCS protocols in clinical settings.

## Introduction

Transcranial direct current stimulation (tDCS) is a non-invasive brain stimulation method that delivers a low-intensity current through the scalp to cortical areas [1]. Several studies have indicated that tDCS applied to frontal areas, implicated with decision-making processes, may have the capacity to attenuate cognitive deficits eminent in various neurological and neuropsychiatric disorders [2, 3]. While tDCS application over the motor cortex has yielded more consistent changes in motor evoked potentials [4, 5], tDCS over frontal regions have varied in its effects on cognitive outcomes [6–8]. This has impacted the progress of using tDCS in the management of cognitive deficits [2]. Although there may be anatomical, physiological and genetic factors contributing to tDCS variability [9], sex-related differences in neural processing and cognitive functions may also be essential to consider.

Sex is a biological characteristic that can influence cognitive functions [10]. Previous studies have revealed that females and males show dissociable abilities in cognitive tasks [11–15]. However, there remains significant debate regarding innate sex differences within cognitive functions, with some studies revealing significant differences [14, 16], while others none [17, 18]. It has been suggested that sex-related influences on cognitive functions may be mediated through sex-linked neurobiological differences [10, 19, 20], including differences in circulating gonadal hormone concentrations, such as estrogen [21], as well as societal and environmental influences [22]. Recently, it has been suggested that sex differences in strategy and outcome assessment, critical aspects of learning, may indirectly drive apparent sex effects on executive functions, rather than innate sex differences in the underlying neurophysiology [23]. This proposal is supported by substantial evidence, including imaging studies that have revealed sex differences in regional brain activity and distinct network activation during task performance [17, 24–30]. Due to possible neuroanatomical substrates contributing to sex differences in cognitive functions—females have a higher percentage of gray matter, while males have a higher percentage of white matter [24]. In the Stop-Signal Task, a commonly used neuropsychological task [28, 31, 32], which simulates a dynamic environment whereby inhibition of inappropriate responses is sometimes required, Gaillard et al. [28] found that although males had better task performance, regional brain activity was attenuated in males in comparison to females in the frontoparietal network, as well as subcortical areas. Interestingly, in another study employing the same task, these sex-related differences in the network underlying cognitive task performance were observed even when there were no sex-dependent behavioural differences [27]. Therefore, it is evident that sex differences in the underlying networks which support cognitive task performance may exist, even in the absence of detectable behavioural differences.

Thus, if there are such differences between females and males in the neural networks underlying cognitive tasks, then the behavioural effects of tDCS may also differ by sex. Indeed, tDCS studies implementing various parameters and stimulation modes in healthy and neuropsychiatric subjects have reported an interaction between cortical modulation and sex [33]. In comparison to males, females often demonstrated more behavioural benefits from the stimulation and heightened cortical excitability [6, 33]. It has been proposed that these sex-related differences in tDCS effects may emerge from non-specific sex factors, such as cranial bone thickness and density, particularly in frontal and parietal regions, leading to females receiving less current than males at cortical areas even when the same current density is applied [34]. In the context of cognitive tasks, the application of tDCS has been shown to enhance emotional recognition [35], search behaviour [36], and theory of mind ability [37, 38] in females but not males [6].

Moreover, in line with the proposal of sex differences in the neural networks underlying cognitive tasks, the laterality of stimulation effect has also been shown to vary between sexes [6]. In females, an enhancement of verbal working memory occurred with stimulation of the right DLPFC, but left DLPFC stimulation in males [6, 39]. These studies suggest that there might be sex-related differences in the outcome of tDCS application. Thus, the application of a uniform tDCS protocol for both sexes may be inadequate, attributing the need for a more thorough understanding of the sex-dependent outcomes of tDCS.

Although numerous studies have examined the influence of tDCS on response inhibition, these studies have not examined nor adequately counterbalanced for sex [8, 9, 40, 41]. Therefore, the issue of sex-dependency of tDCS effects, particularly on executive control, remains unknown. Executive control [42] refers to a set of higher-order functions that facilitate goal-directed behaviour by optimizing the flexible use of limited cognitive resources [42, 46–48]. Such control enables the exclusion of irrelevant and distracting stimuli and prioritizes task-relevant information enabling adaptive behaviour in a changing environment [42, 46–48]. An essential facet of executive control is response inhibition, which enables the suppression of inappropriate or no-longer relevant actions [43, 44] and is facilitated through prefrontal cortical regions, including the inferior frontal gyrus (IFG) and the DLPFC [44]. Previous studies have observed sex-related differences in inhibition ability [6, 26–29, 45]. We have previously demonstrated in the context of the Stop-Signal Task that following practice, females learned to improve their response inhibition ability to a greater extent than males [45].

Background acoustic stimuli, particularly music, are commonly experienced contextual factors [49] and can modulate inhibition ability in executive control tasks [45, 50]. Past research examining the influence of music has been inconsistent, revealing that music may increase [51, 52] or decrease [53, 54] performance in cognitive, perceptual, and motor tasks [45]. Specifically, in the context of cognitive tasks, the modulatory effects of music may be mediated by the alteration of activation levels in brain areas presumably involved in executive functions, such as the DLPFC [55]. Furthermore, the behavioural influence of music may also differ between sex [45, 49, 56]. In our previous study, we observed that background music, introduced as a contextual factor, had a sex-dependent influence on participants' response time, whereby females' response time was attenuated by music and males response time increased [45].

Previous studies have indicated that anodal tDCS can modulate response inhibition and response execution in the Stop-Signal Task [7–9, 40]. We have previously reported that anodal tDCS and music interactively influence response inhibition and practice-related learning (a behavioural improvement between testing sessions) [57, 58]. High-tempo music diminished practice-related changes in inhibition ability; however, these practice-related changes were reinstated by anodal tDCS applied to the DLPFC [58]. TDCS applied to the DLPFC [41], or the inferior frontal gyrus (IFG) [7, 8] have been shown to improve response inhibition ability. TDCS applied to the DLPFC has also been shown to augment behavioural adjustments following error commission [32]. Anodal tDCS applied to the IFG has shown mixed effects in influencing the response execution in Go trials; with some studies reporting increased response times [9] 84, and others reporting decreased response times [85]. However, it remains unclear whether these effects were sex-dependent. Therefore, in this study, we investigated whether contextual factors such as background acoustic condition and participants' sex interactively influence the effects of tDCS on executive functions, specifically, response inhibition and execution.

Past research has indicated variabilities in the cognitive outcomes of tDCS [2] and music [51–54]. Although the factors contributing to such variabilities remain unclear [9], one contributing factor may be sex, as sex-linked neurobiological differences [10, 19, 20] may influence the neural network underlying performance in cognitive tasks [17, 25–30]. In line with this proposal, sex-dependent influences of music on cognitive functions have been reported [45, 49, 56]. However, as mentioned previously, a majority of previous studies did not adequately counterbalance for sex [8, 9, 40, 41, 59]. Thus, the sex-dependency of tDCS effects on executive functions and its interaction with background music remains unknown.

## Methods

### Study design

In the current study, we aimed to assess whether there is a sex-dependent influence of tDCS on inhibition ability in the Stop-Signal Task. Participants completed the Stop-Signal Task before (pre-tDCS) and after (post-tDCS) tDCS application. Either anodal or sham tDCS of the left DLPFC was administered in two sessions (one-week washout period) [57]. We selected left DLPFC for stimulation because previous imaging studies have shown activation of bilateral DLPFC in the context of the Stop-signal task [57, 60–62]. We also assumed that tDCS would induce neuroplasticity in the neural networks that support action selection and action inhibition and therefore targeted the contralateral hemisphere of the responding hand. Therefore, all right-handed subjects were recruited for this study and they used their dominant hand for delivering responses. During the task performance, participants were exposed to one of three background acoustic conditions (high-tempo music, low-tempo music, and no-music).

Critically, to ascertain whether there was a sex-dependent influence of tDCS in the context of the Stop-Signal Task, the participant's sex had to be counterbalanced fully across all conditions. To achieve this, participant's sex was counterbalanced across (1) stimulation conditions (either anodal or sham), (2) music condition (high-tempo music, low-tempo music and no-music), and (3) the order in which conditions were presented (e.g. anodal stimulation in the first week and sham stimulation in the second week, or sham stimulation in the first week and anodal stimulation in the second week).

### Participants

73 right-handed participants (37 females, 18–32 years old) joined this study. Priori power analysis [63] was conducted using GPower [64] to compute the required sample size. Considering an effect size of 0.21 for practice-related learning in the Stop-Signal Task (observed in a previous study) [32], alpha at 0.05, and power at 0.80, the estimated minimum sample size required to detect this effect was 36 participants. However, to achieve complete counterbalancing across sex, simulation type, music condition, and order, more participants were recruited. Handedness was confirmed using the Edinburgh Handedness Inventory [65]. Any participant with a self-reported current or history of neurological/neuropsychiatric conditions was excluded. All participants gave written consent before their involvement, and the study was approved by the Human Research Ethics Committee of Monash University and conformed to the World Medical Association Declaration of Helsinki.

### Apparatus

**Fig. 1:**
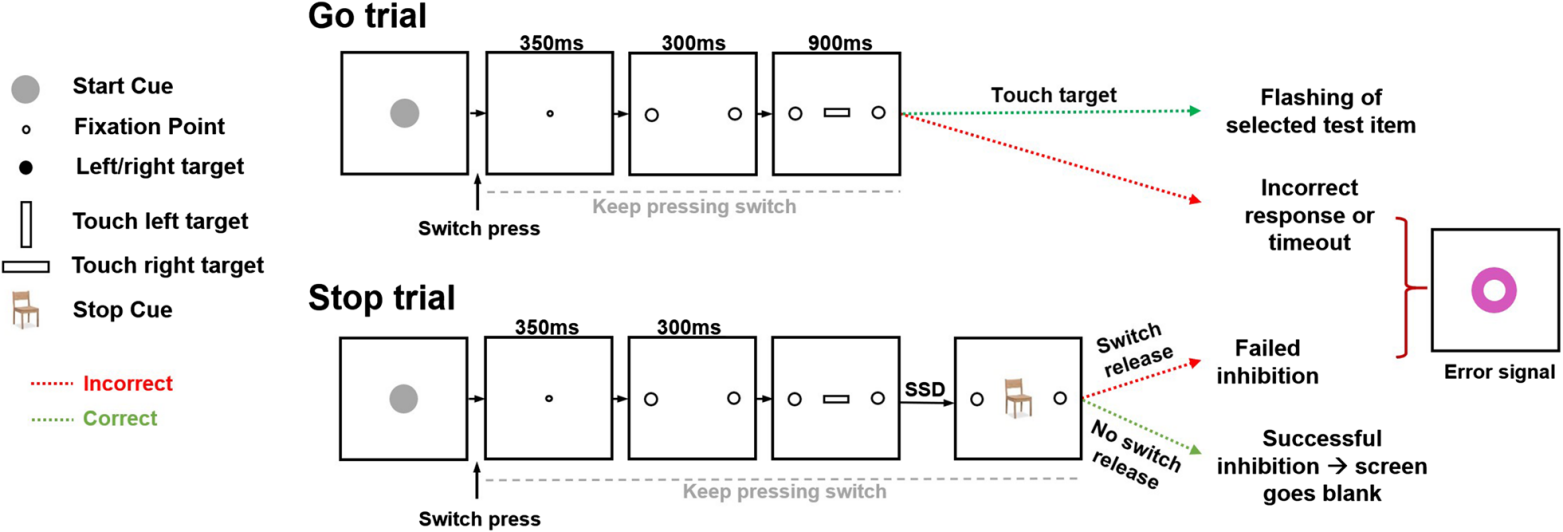
Stop-Signal Task. In Go trials, a start cue instructs participants to press and hold a switch with their right index finger. The start cue is then replaced by a fixation point (for 350 ms) before two target items appear (for 300 ms) to the right and left of the fixation point. A go-cue, a white bar, then replaces the fixation point. If the bar is horizontal or vertical, participants were to select (by touch) the right or left target item, respectively, within a 900 ms time window. If the correct target was not selected within the time window, an error-signal was shown, and the trial was considered as an error. Within Stop trials, events were the same until go-cue onset, however, following a variable delay, a stop signal (multi-coloured image) replaced the go-cue. This stop-signal (multi-coloured images of various objects) instructed participants to inhibit their response and keep pressing the switch. Switch release was considered as an error in Stop trials

Participants completed a computerized version of the Stop-Signal Task (Fig. 1) in sound-attenuated rooms and responded using a touch screen (3 M™ MicroTouch™) and a switch centred at the base of the screen. Stimulus presentation and data acquisition were controlled by CORTEX (National Institute of Mental Health) at 1000 Hz. Before the first testing session, participants read an instruction statement explaining the task and requirements and received pre-defined verbal instruction.

### Behavioural task

The computerized Stop-Signal Task, shown in Fig. 1, has been reported and validated in previous studies [32, 46, 57]. The Stop-Signal Task requires the completion of two randomly intermingled trial types: Go (70% of trials) and Stop (30% of trials) trials. In a Go trial, a start-cue instructed participants to press and hold the switch. If the switch remained pressed, a fixation point replaced the start cue, and 350 ms later, target items (white circles) appeared to the right and left of the fixation point and remained on the screen for 300 ms. A vertical or horizontal white bar (go-cue) then replaced the fixation point, signalling participants to release the switch and touch the left or right target item, respectively. Participants were instructed to use only their right index finger for both pressing the switch and touching the screen, and had a limited time window from the presentation of the go-cue to touch the screen (900 ms). If the correct target was selected within the time window, visual feedback was provided (the selected target item would flash off (200 ms) and on (200 ms)). However, if the wrong target was selected, the response was outside of the time window, or the switch was released early, all items disappeared, and an error signal was presented. Trial events in Stop trials were the same as in Go trials. However, following a variable delay, a stop signal (a multi-coloured image) replaced the go-cue. Each stop signal (multi-coloured image) was only presented once per participant per session. The stop signal signified participants to inhibit their initiated response, thus continue pressing the switch. The delay (stop signal delay: SSD) between the go-cue onset and the stop-signal onset was altered in a performance dependant step-wise manner, if the previous Stop trial was correct, the delay would increase a step (40 ms), whereas if the previous Stop trial was erroneous (failed inhibition), it would decrease a step (40 ms). In the first stop trial, SSD was 15 ms. This step-wise adaptive procedure alters the difficulty of inhibition in Stop trials; therefore, ~ 50% accuracy in Stop trials is anticipated.

To ensure participants understood task requirements before data collection commenced, they completed a practice block that contained only Go trials, and participants had to complete 16 correct trials across 20 consecutive trials to enter the data collection block. This data collection block in each pre- and post- tDCS testing was performance-based and ran until 250 correct trials (including both Stop and Go trials) were completed. To mitigate the influence of non-specific factors such as arm length or muscle mass, we considered the time between the onset of the go-cue and the initial movement (switch release) as the response time.

### TDCS protocol

Participants completed the Stop-Signal Task before and after tDCS administration in pre- and post- tDCS testing, respectively [46, 57]. During a silent rest period between testings, either anodal or sham tDCS was administered. Electrode positioning was localized using the international 10–20 system [66]. A 2.5 × 4 cm saline-soaked multi-use carbon rubber electrode with sponge pockets was placed over the left DLPFC (F3, international 10–20 system), and a larger, 4 × 6 cm, reference electrode over the right supraorbital area [3, 32, 46, 57, 67]. In the anodal condition, direct current was applied at 1.5 mA for 10 min using a tDCS device (Intelect® Advanced Therapy System, Chattanooga, USA). To blind participants in the sham condition, the conventional approach of applying a transient current (15 s fade in, 30 s constant at 1.5 mA, and 15 s fade out) was implemented [3, 46, 67]. During stimulation, participants were required to rate the subjective experience of any side effects (e.g. burning, headache, tingling, itching and pain) on linear numeric scales (e.g., 0 = side effect not present to 10 = side effect worst imaginable)— no significant or adverse reactions were reported. The post-tDCS testing commenced 5 min from the cessation of tDCS application. During this time, electrodes and attachments were detached, and participants could adjust their glasses/headwear. All participants completed two sessions and received both stimulation types (anodal and sham), separated by a washout period (1 week). The order in which participants received stimulation type was counterbalanced.

### Background acoustic conditions

In this study, background acoustic conditions were varied to examine whether they influenced the effects of tDCS. Participants were pseudo-randomly assigned into one of three background acoustic conditions (no-music, high-tempo music, low-tempo music). The background audio conditions were played using wireless headphones while participants completed the task (during both pre- and post- tDCS testing). We have previously reported the influence of these background acoustic conditions and their interaction with tDCS on cognitive functions [46].

### Data analyses

The exclusion of any data points requires applying arbitrary criteria, and therefore, we used all collected data points without the removal of outliers. In Go trials, the response time (RT) was measured as the time between the onset of the go-cue and switch release.

To ascertain whether there was a sex-dependent influence of tDCS on Stop-Signal Task performance, analyses were conducted for each behavioural measure using a repeated-measures ANOVA. For each pre- and post-tDCS session, a mean was calculated for each measure in each participant. Each ANOVA contained 4 factors; two within-subject factors: Stimulation Type (Anodal/ Sham) and Practice (Pre-tDCS/ Post-tDCS), and two between-subject factors: Sex (Female/ Male) and AcousticCondition (no-music/ high-tempo music/ low-tempo music). Within this structure, a significant two-way interaction between Stimulation Type and Practice would indicate that tDCS modulated practice-related learning (the behavioural change between the pre- and post- tDCS testing). A three-way interaction between StimulationType, Practice and Sex, would indicate a sex-dependent influence of tDCS on practice-related learning. In all analyses, sphericity was confirmed using Mauchly's test, and where necessary, a Greenhouse–Geisser correction was applied. Partial Eta Squared is reported for all significant effects and indicates the proportion of the total variance which the effect can explain.

## Results

### Response inhibition

#### Percentage of correct responses in stop trials

In each Stop trial, the delay of the stop signal after the go-cue (SSD) was adjusted in a performance dependant step-wise manner to alter the difficulty of successful inhibition [31] (see Methods) so that an accuracy level of ~ 50% was expected in Stop trials. Stop trial accuracy was 55.4 ± 0.45 (mean ± SE) and 55.3 ± 0.51 in the sham and anodal tDCS condition, respectively. Moreover, a multi-factor ANOVA: StimulationType [tDCS/ Sham, within-subject factor] × Practice [Pre-tDCS/ Post-tDCS, within-subject factor] × Sex [Female/ Male, between-subject factor] × AcousticCondition [No-music/ High-tempo/ Low-tempo, between-subject factor], applied to the percentage of correct responses in Stop trials showed no significant main effect or interactions between tDCS, Practice, and Sex (all *p* > 0.1), indicating that even though the accuracy was slightly above 50%, the adaptive procedure was effective in maintaining Stop trial accuracy around 50% for all conditions.

#### There was no sex-dependent effect of tDCS on inhibition ability

**Fig. 2:**
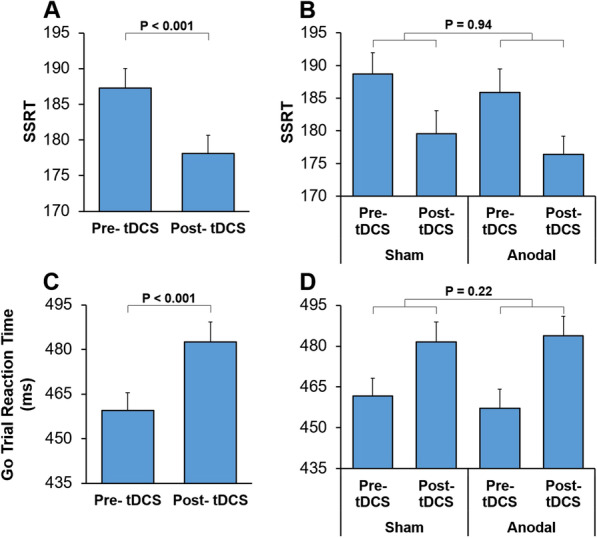
Practice related changes in response inhibition and response execution. **A** SSRT, an index of response inhibition ability, is shown for the pre- and post- tDCS testing. A shorter SSRT indicates a better inhibition ability. SSRT decreased in post-tDCS sessions, indicating practice-related learning. **B** SSRT is shown for the pre- and post- tDCS testing for each tDCS condition (sham or anodal). The observed practice-related learning was not influenced by tDCS condition. **C** Response time in correct Go trials is shown for the pre- and post- tDCS testing. Response time increased in post-tDCS sessions, indicating practice-related slowing. **D** Response time in correct Go trials is shown for the pre- and post-tDCS testing for each tDCS condition (sham or anodal). The observed practice-related learning was not influenced by the tDCS condition. Error bars in all figures show the standard error of the mean. In **B** and **D** The p-value indicates the significance level for the interaction of StimulationType and Practice

Stop signal reaction time (SSRT) is a reliable estimation of the participants' inhibition ability. Recent studies have proposed that the most appropriate way to derive SSRT is via the 'integration method' [31, 68]. We used the integration method for estimation of SSRT and therefore considered the *n*th Go trial in RT distribution, where *n* is equal to the percent of failed response inhibition (e.g. if a participant had 47% accuracy in Stop trials, the 53rd percentile of the Go RT distribution would be used). A shorter SSRT indicates a better response inhibition ability [44, 57]. A multi-factor ANOVA: StimulationType × Practice × Sex × AcousticCondition, was applied to SSRT. The main effect of Practice was significant (F(1, 67) = 16.17; *p* < 0.001) (Partial Eta Squared = 0.19) (Fig. 2A), however there was no significant interaction between Practice and tDCS. These indicate a practice-related improvement in inhibition ability, reflected as a decreased SSRT in the post-tDCS session in both sham and anodal conditions (Fig. 2B). The interaction of Practice and Sex (F(1,67) = 1.40; *p* = 0.24) was not significant (F(1, 67) = 0.005; *p* = 0.94). The interaction between Practice, StimulationType, and Sex factors (F(1,67) = 2.49; *p* = 0.12) was not significant either. These results indicate that the practice-related improvement in inhibition ability was not influenced by sex or a sex-dependent tDCS effect.

#### There was no sex-dependent interaction between tDCS and background acoustic conditions on inhibition ability

The ANOVA (StimulationType × Practice × Sex × AcousticCondition) applied to SSRT, also showed that the main effect of AcousticCondition was not significant (F(2,67) = 1.05; *p* = 0.36). The interaction between AcousticCondition and Sex was not significant (F(2,67) = 0.69; *p* = 0.51), indicating that background acoustic condition did not exert a sex-specific influence on inhibition ability (Fig. 4A). The interaction between AcousticCondition, StimulationType, and Practice factors was significant (F(2,67) = 3.50; *p* = 0.04) (Partial Eta Squared = 0.09) (as previously reported [46]), however there was no significant interaction between tDCS, Practice, AcousticCondition, and Sex factors (F(2,67) = 0.36; *p* = 0.70), indicating that the interactive effects of background acoustic condition and tDCS on inihbition ability was uniform in males and females.

### Response execution

#### There was sex-dependent effects of tDCS on percentage of correct responses in Go trials

**Fig. 3:**
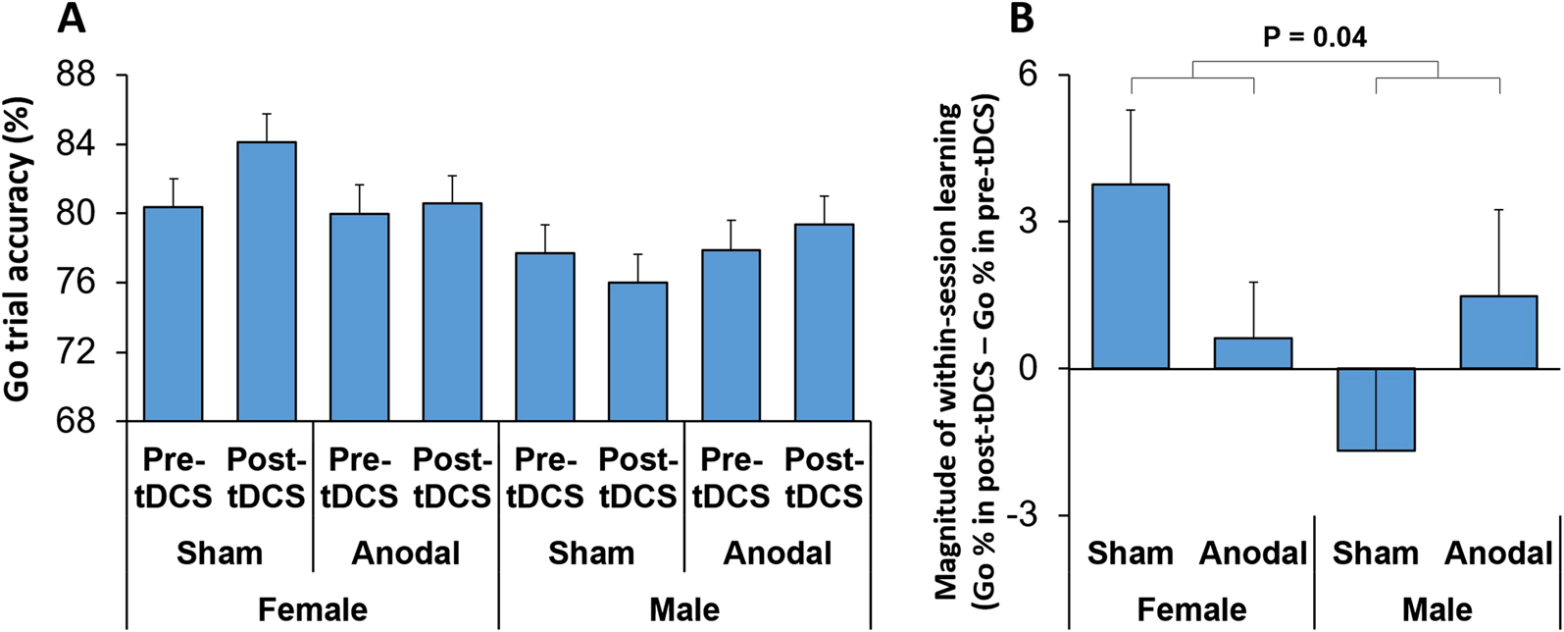
Sex-dependent effects of the tDCS on response execution. **A** Percentage of correct responses (accuracy) in Go trials is shown for pre- and post- tDCS testing for each stimulation type (Anodal or Sham) separated by sex. To ease visual comparison **B** shows the difference between the pre- and post- tDCS testing in Go trial accuracy (magnitude of within-session learning) for each stimulation type separated by sex. The magnitude of practice-related learning was attenuated by tDCS in females, while in males, tDCS reversed the direction of practice-related changes. The p-value indicates the significance level for the interaction of StimulationType, Practice, and Sex factors

To examine whether tDCS, background acoustic condition, or participants' sex influenced accuracy in response execution, a multi-factor ANOVA: StimulationType × Practice × Sex × AcousticCondition, was applied to the percentage of correct responses in Go trials. The main effect of Sex was significant (F(1,67) = 4.12; *p* = 0.046) (Partial Eta Squared = 0.06), indicating that Go trial accuracy differed between sexes. Females had higher accuracy (81.34% ± 1.27 (Mean ± standard error)) than males (77.69% ± 1.29). The main effect of Practice was not significant (F(1,67) = 1.64; *p* = 0.21). However, there was a significant interaction between StimulationType, Practice, and Sex factors (F(1,67) = 4.19; *p* = 0.04) (Partial Eta Squared = 0.06). This significant interaction indicates that the effects of tDCS on the accuracy of response execution was sex-dependent (Fig. 3A, B). To further assess this 3-way interaction, we calculated the difference between the pre- and post-tDCS testing, which indicated the magnitude of practice-related learning in Go trial accuracy. The practice-related change (improved accuracy observed in the sham session) was attenuated in females by anodal tDCS. In contrast, in males, tDCS reversed the direction of practice-related learning from decreased accuracy in sham sessions to enhanced performance in anodal sessions (Fig. 3B).

#### There was no interaction between background acoustic conditions and paticipants’ sex in modulating performance in Go trials

**Fig. 4:**
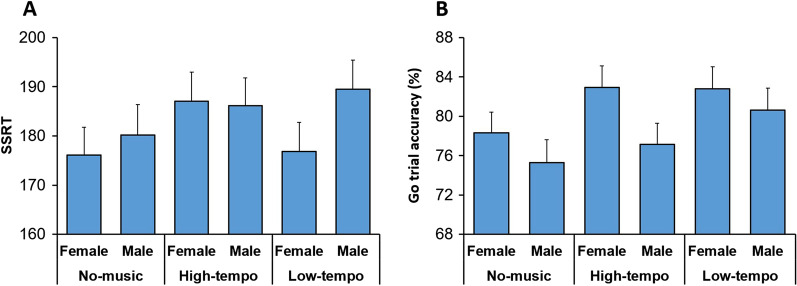
Background acoustic conditions did not exert sex-dependent effects. **A** Percentage of correct responses (accuracy) in Go trials is shown for each acoustic condition (No-music, High-tempo, Low-tempo) separated by sex. Accuracy was not modulated by the background acoustic conditions in a sex-dependent manner. **B** SSRT is shown for each acoustic condition (No-music, High-tempo, Low-tempo) separated by sex. SSRT was not modulated by the background acoustic conditions in a sex-dependent manner

The ANOVA (StimulationType × Practice × Sex × AcousticCondition) applied to the percentage of correct responses in Go trials, also showed that the main effect of AcousticCondition was not significant (F(2,67) = 2.54; *p* = 0.09), and its interaction with other factors (all *p* > 0.20) were not significant. Specifically, the interaction between AcousticCondition and Sex factors was not significant (F(2,67) = 0.69; *p* = 0.51), indicating that background acoustic condition did not exert a sex-specific influence on the accuracy of response execution (Fig. 4B). Moreover, there was no significant interaction between tDCS, Practice, AcousticCondition, and Sex factors (F(2,67) = 0.11; *p* = 0.90), indicating that there was no sex-dependent interactive effect of background acoustic condition and tDCS on the accuracy of response execution.

#### There were no sex-dependent effects of tDCS on participants’ response time

Response time (RT) in Go trials reflects the participants' speed of response initiation after the onset of the go-cue and was calculated as the time between go-cue onset and the release of the switch [46, 57]. A multi-factor ANOVA: StimulationType × Practice × Sex × AcousticCondition, was applied to RT in correct Go trials. There was a significant main effect of Practice F(1, 67) = 43.65; *p* < 0.001) (Partial Eta Squared = 0.39), indicating that following practice, RT increased in the post-tDCS testing (practice-related learning; Fig. 2C). Such an increase in RT following practice in the stop-signal task has been reported previously [69] and presumably reflect anticipation of stop-cue and proactive slowing to decrease the likelihood of error in response inhibition [70]. The interaction between StimulationType and Practice factors (F(1,67) = 1.54; *p* = 0.22) was not significant, indicating that the application of tDCS did not influence the practice-related proactive slowing (Fig. 2D). The main effect of Sex was not significant (F(1,67) = 0.41; *p* = 0.53) either, indicating that Go trial RT did not differ between males and females. Importantly, the interaction between StimulationType, Practice, and Sex was not significant (F(1,67) = 0.44; *p* = 0.51), indicating no sex-dependent influence of tDCS on proactive slowing.

Previous studies [32] have reported response slowing in those Go trials, which were preceded by a failed inhibition (error) in the preceding Stop trials. To assess whether such post-error slowing was sex-dependently modulated by the tDCS, we classified the trials to those correct Go trials, which preceded by another correct Go trial (GcGc; c = correct, G = Go trial) and those correct Go trials, which were preceded by a failed inhibition in the preceding Stop trial (SeGc; e = error S = Stop trial). Then, we applied a four-way ANOVA: (Post-error [SeGc/GcGc, within-subject factor] × Stimulation × Practice × Sex, to the RT in the second trial of SeGc and GcGc trial sequences. There was a significant main effect of Post-error (F(1, 71) = 535.89; *p* < 0.001) (Partial Eta Squared = 0.88), indicating that RT increased following a failed Stop trial. There was a significant main effect of Practice (F(1, 71) = 535.89; *p* < 0.001) (Partial Eta Squared = 0.88), indicating that following practice, RT increased in both trial sequences. The main effect of Stimulation (*p* = 0.80), or Sex (*p* = 0.63) was not significant. Moreover, there was no significant interaction between Sex and other factors indicating that sex did not influence post-error slowing.

The ANOVA also showed that the main effect of AcousticCondition (F(2,67) = 1.16; *p* = 0.32) and its interaction with other factors (all *p* > 0.15) were not significant. These indicate that the background acoustic conditions did not influence RT or proactive slowing.

## Discussion

### The effects of tDCS on inhibition ability were not different between males and females

SSRT is a measure of response inhibition that assesses an individual's ability to suppress prepotent responses where a shorter SSRT indicates a better inhibition ability. Although some studies have reported that response inhibition can be modulated by the application of tDCS [7, 8], the sex dependency of these effects has remained unclear partly because full counterbalancing for participants' sex was not considered. Table 1 highlights study design variability and a prominent lack of sex counterbalancing in past studies. Thus, it has been difficult to draw inferences regarding the sex dependency of the tDCS effects on response inhibition or execution in the context of stop-signal tasks. In our study, participants' sex was fully counterbalanced across all conditions and therefore provided an opportunity to examine the sex dependency of tDCS effects on response inhibition and response execution. Our results indicate that although tDCS showed interaction with background acoustic condition and modulated inhibition ability, there were no interactive effects between tDCS and sex or between background acoustic condition and sex on inhibition ability. However, the effects of tDCS on response execution was dependent on participants' sex.

**Table 1:** Summary of methodological variabilities and sex-related effects in previous tDCS studies examining response inhibition

Study	Stimulation	Brain Region	*n*	Males	Sex counterbalanced?	Intensity (mA)	Duration (min)	Online/offline	Task	Study design	Main findings on inhibitory ability	Main findings on sex-related modulation
[83]	Anodal/ Cathodal	SMFC	18	6	No	0.7	9	Offline	SST	Single-blinded, crossover, sham-controlled, pre- and post- tDCS testing	No modulatory effect	Not reported
[40]	Anodal	rPFC	14	3	No	1.5	15	Online	SST	Single-blinded, between-group, sham-controlled	Reduced SSRT	Not reported
[84]	Anodal	rIFC	22	4	No	1.5	18	Online	SST	Single-blinded, crossover, sham-controlled	Reduced SSRT	Not reported
[85]	Anodal	rIFC	13	7	Yes	1.5	20	Online	SST	Single-blinded, crossover, sham-controlled	No modulatory effect	Not reported
[9]	Anodal	rIFG	22	6	Yes	1.5	15	Offline	SST	Between-group, post-tDCS testing	Reduced SSRT	Not reported
[57]	Anodal	lDLPFC	73	36	Yes	1.5	10	Offline	SST	Single-blinded, crossover, sham-controlled pre- and post- tDCS testing	Reduced SSRT	Not reported
[86]	Anodal	OFC	45	16	No	1.5	30	Offline	SST	Single-blinded, between-group, sham-controlled, pre- and post- tDCS testing	No modulatory effect	Not reported
[41]	Anodal	rDLPFC	59	21	Yes	0.5	19	Offline	SST	Single-blinded, between-group, sham-controlled, pre- and post- tDCS testing	Reduced SSRT	Not reported
[87]	Cathodal	rDLPFC	45	12	No	0.5	20	Offline	SST	Single-blinded, between-group, sham-controlled, pre- and post- tDCS testing	Increased SSRT	Not reported
[88]	Anodal	IFC	52	N.S	Yes	1	20	Offline	SST	Between-group, pre- and post- tDCS testing	Reduced SSRT	Not reported
[7]	Anodal	rIFG	11	3	No	1	10	Offline	SST	Crossover, sham-controlled, post-tDCS testing	Reduced SSRT	Not reported
[59]	Anodal	pre-SMA and M1	40	22	No	1	10	Offline	SST	Crossover, sham-controlled, pre- and post- tDCS testing	Reduced SSRT	No effect
[46]	Anodal	DLPFC	73	36	Yes	1.5	10	Offline	SST	Single-blinded, crossover, sham-controlled pre- and post- tDCS testing	Reduced SSRT	Not reported
[89]	Anodal	rIFC	30	14	Yes	1.5	20	Offline	SST	Single-blinded, between-group, sham-controlled, pre- and post- tDCS testing	Reduced SSRT	Not reported
[90]	Anodal/ Cathodal	IFG	72	18	No	1.5	20	Online	SST	Single-blinded, between-group, sham-controlled	No modulatory effect	Not reported
[8]	Anodal/ Cathodal	rIFG	115	29	No	1.5	20	Offline	SST	Single-blinded, between-group, sham-controlled, post-tDCS testing	Reduced SSRT with anodal stimulation	Not reported

r or l before brain region denotes right and left, respectively. Abbreviations: N.S, not specified; SMFC, superior medial frontal cortex; PFC, prefrontal cortex; IFC, inferior frontal cortex; OFC, orbitofrontal cortex; IFG, inferior frontal gyrus; pre-SMA, supplementary motor area; M1, primary motor cortex

### Response time in Go trials was not modulated by tDCS

In the context of stop-signal tasks, participants are instructed to respond as fast and accurately as possible. However, previous studies have consistently reported a response slowing following practice [69]. This slowing has been described as a learning-induced proactive strategic adjustment where the subject balances possible 'going' and 'stopping' by slowing their response to better anticipate and inhibit the response if the Stop signal is shown [70]. In line with past literature [69], we also observed such practice-related slowing in response execution (Go trials; Fig. 2C, D). However, this practice-related learning was not modulated by applying tDCS over the DLPFC and was not sex-dependent. Previous studies examining the effects of tDCS on response time have led to contradictory outcomes; with some studies showing modulation of response time when bilateral anodal or cathodal tDCS was applied to the superior temporal sulcus [71] or motor areas [72, 73], however, in line with our results, some other studies did not observe any modulation of response time by tDCS application [74, 75]. These differences might reflect differences in the required task, electrode montage and therefore stimulated area, or other contextual factors.

### Accuracy in Go trials was modulated by tDCS in a sex-dependent manner

We found that the percentage of correct responses (accuracy) in Go trials was influenced by tDCS in a sex-dependent manner (Fig. 3). The difference in accuracy between pre-tDCS and post-tDCS testing was attenuated in females by the application of tDCS. However, this difference changed its direction in males following tDCS application (Fig. 3B). This finding suggests that tDCS over DLPFC influences learning in response execution in a sex-dependent manner.

The underlying neural processes still remain unknown however we assume that practice-related neuroplastic changes occur in neurocircuitries and manifest as improved or decreased accuracy in females and males, respectively. The affected neurocircuitries might differ between males and females, however these learning-related neuroplastic changes might depend on some underlying neural mechanisms, which are also affected by brain stimulation (tDCS) and normally lead to neuroplasticity following tDCS (i. e. learning-related and tDCS-related neuroplasticity might depend on overlapping neural substrate and underlying mechanisms) Therefore, tDCS might have played a modulatory role in modifying practice-related neuroplasticity, but led to opposite outcome in males and females.

Boggio et al. [35] applied anodal tDCS to the left temporal cortex in a go-no-go task, which required differentiation of facial expressions to determine the requirement for either response execution or inhibition. Females made fewer errors following anodal stimulation compared to sham stimulation when responding to sad faces. Whereas the opposite influence was observed in males in that anodal stimulation increased the error rate when responding to sad faces [35]. Although the tDCS-induced changes appeared in the opposite direction to that of our findings, differences in the stimulated region and the requirement for detection of emotional information for guiding behaviour might have influenced the sex-related differences. Nevertheless, these findings indicate a sex-dependent influence of tDCS on response execution which appears across various tasks.

It has been previously reported that response execution in Go trials is akin to motor responses in speeded reaction tasks [74]. Within this domain, previous studies have indicated that learning and tDCS may interact to modulate performance in tasks that involve precise or timely ordered motor functions [76–78]. In a recent study, Horvath et al. [74] proposed that variation of three common factors contribute to the heterogeneity of tDCS influence in speeded reaction tasks: 'current density', 'reference electrode placement', and 'stimulation timing'. However, in a systematic manipulation of these factors to assess their influence, no predictable tDCS influence could be determined [74]. Thus, we infer that differences in the outcome of tDCS in past findings cannot solely be attributed to differences in stimulation parameters (such as electrode montage, intensity, duration) and other factors, such as sex-related differences in strategy and the involved neural networks, may contribute to the modulatory effects of tDCS.

Moreover, the sex dependency of tDCS effects observed in our study, were not merely due to a sex-dependent shift in the speed-accuracy trade-off. The speed-accuracy trade-off explains a relationship between response speed and accuracy; so that as the speed of responding increases, the accuracy rate decreases [79–81]. Our findings indicate that the sex-dependent effect of tDCS on response execution accuracy (Fig. 3) was not accompanied by a concomitant sex-dependent tDCS effect on response time. However, the accuracy of response execution might depend on separate neural mechanisms that are sex-dependently modulated by direct current stimulation of the DLPFC. Furthermore, in a recent study, Thomas et al. [82] quantified the effects of sex-related morphological changes on the tDCS-induced cortical electrical field (EF). Their results indicated differences in gray and white matter distribution between sexes, and the induced cortical EF was higher in females than males [82]. These neuroanatomical and physiological differences might also bring insight to the neural substrate of sex-dependency of tDCS effects.

Neuroimaging studies employing various cognitive tasks for assessing inhibitory control have revealed that females and males show distinctive activation of brain regions, indicating sex-specific involvement of brain networks in inhibitory control. Li et al. [27] examined whether there were any sex differences in the neural substrates of response inhibition during the Stop-Signal Task. Although there was no difference in behavioural measures between males and females, functional magnetic resonance imaging revealed that males exhibited activation in medial superior frontal and anterior cingulate cortices in Stop trials when response inhibition was required. In contrast, females exhibited activation in the caudate tail, demonstrating distinct sex-differences in regional brain activation in the context of the Stop-Signal Task [27]. Such differences in regional brain activation during the Stop-Signal Task performance have been confirmed in other imaging studies [28]. However, using a fully counterbalanced study to examine the sex-dependency of tDCS over DLPFC, we did not observe any sex dependency in the effects of tDCS or in the interaction of other factors in modulating inhibition ability. In our study, the tDCS intensity and electrode montage over DLPFC effectively showed the interaction of acoustic condition and tDCS in modulating inhibition ability [46]; however, these effects were not sex-dependent.

Our findings indicate that the effects of tDCS on the accuracy of response execution were dependent on the participants' sex. Specifically, the direction of pre-tDCS to post-tDCS changes was different between males and females (Fig. 3). This highlights that in the stop-signal task, the sex dependency of tDCS effects is specific to neural processes that mediate response execution. Such specificity in tDCS effects has also been observed in past literature examining different executive control processes, including stimulus discrimination, working memory, and risk-taking behaviours [6, 35–37, 39]. Furthermore, as the sex dependency of tDCS effect on action execution was not accompanied by alterations of response time, these differences could not be attributed to sex-specific strategic adjustments (e.g. speed-accuracy trade-off).

The sex-dependent modulatory role of tDCS on accuracy of Go trials brings some insights for interpreting the lack of tDCS effects on inhibition ability. The interaction of Practice and Sex was not significant when the ANOVA was applied on the SSRT. This means that there were no sex-dependent differences in practice-related changes in SSRT. We also found that there was no interaction between tDCS and Sex factors for response inhibition (no interaction for percentage of correct response in Stop trials and no interaction for SSRT). Therefore, it is possible that when there were sex-related differences in learning (practice-related differences between males and females as we observed for accuracy in Go trials), tDCS might have been effective in sex-dependently modulating such learning-related effects. However, tDCS did not have a uniform effect in males and females: it prevented practice-related changes in females but enhanced practice-related changes in males. When there were no sex-related differences in learning (as we observed for SSRT), tDCS could not exert any sex-dependent modulatory effects. This further suggests that neuroplasticity induced by tDCS might depend on neurocircuitries and mechanisms that naturally mediate the practice-related plasticity in males and females.

Thus, we propose that, in line with neuroimaging studies [27, 28], there might be significant sex-related differences in the underlying neural mechanisms of response execution but not the response inhibition. Our findings suggest that these sex-related differences might provide different susceptibility to direct current stimulation of the DLPFC. These sex-dependent tDCS effects may arise from sex-related anatomical differences [82]. Our findings provide evidence for sex-dependent effects of tDCS. However, it is difficult to directly compare our findings with the previous tDCS studies on inhibition ability because in many of these studies, the sex dependency of tDCS effects could not be reliably assessed due to inadequate counterbalancing of participants’ sex or a lack of cross-over design (see Table 1).

Future studies need to adequately control for participants' sex, as sex has the potential to influence the tDCS effect. Sex-related differences in the activation of neural network in the context of cognitive tasks might alter the susceptibility of neural networks to the brain stimulation effects (neuroplasticity) and lead to different outcomes in males and females following tDCS application. The combination of neuroimaging techniques such as concurrent tDCS with electroencephalography or tDCS with functional magnetic resonance imaging might help to delineate the task-relevant neural circuits (networks) before application of tDCS; and also differential alterations in the activation of these circuits following tDCS application.

### Perspectives and significance

TDCS has been considered as a viable approach to address learning impairment and cognitive deficits in various neurological and neuropsychiatric disorders, however huge variabilities have been observed in its’ cognitive effects, which might be related to contextual factors such as participants’ sex. A majority of previous brain stimulation studies have not counterbalanced the participants’ sex across experimental conditions and therefore the sex dependency of tDCS effects remain unclear. Here, in a sham-controlled cross-over study in which participants’ sex was fully counterbalanced across all experimental conditions, we demonstrate that the effects of tDCS on inhibition ability is uniformly seen in both males and females. However, the effects of tDCS on response execution differs between males and females. These findings have important implications for future research, highlighting the need to adequately control for participants' sex. Furthermore, from a clinical perspective, a better understanding of the sex-related factors contributing to the variability in the cognitive outcomes of tDCS will pave the way for tailoring and implementing sex-specific protocols in the application of tDCS for addressing cognitive deficits in neuropsychiatric and addiction disorders.

## Data Availability

All data generated or analyzed during this study are included in this published article. Raw data may be provided via direct contact with the corresponding author.
